# Supplementary material for: Dietary Patterns and Prevalent NAFLD at Year 25 from the Coronary Artery Risk Development in Young Adults (CARDIA) Study
Source: Nutrients. 2022 Feb 18;14(4):854. doi: 10.3390/nu14040854 (PMC8878386; doi:10.3390/nu14040854)
Supplement: Supplementary file 1 [file nutrients-14-00854-s001.zip › nutrients-1592003-supplementary.pdf]

Supplemental Table S1: Comparison of included and excluded participants based on Year 25 exam attendance (N = 3498)

	**Included** N **= 1726**	**Excluded** N **= 1772**	***p*-value**
Age (year), mean (SD)	50.16 (3.59)	50.16 (3.68)	0.99
Women (%)	998 (57.82)	983 (55.47)	0.16
White (%)	1031 (59.73)	827 (46.67)	<0.0001
Socioeconomic status			
Highest grade completed, mean (SD)	15.97 (2.51)	15.28 (2.59)	<0.0001
Income >$50,000/year (%)	1229 (71.21)	1012 (59.04)	<0.0001
Access to medical care			
Report regular medical care (%)	1598 (92.58)	1588 (89.62)	0.002
Difficulty accessing healthcare (%)[1]	169 (9.79)	229 (13.11)	0.002
BMI (kg/m^2), mean (SD)	29.62 (6.74)	30.71 (7.60)	<0.0001
Obese, BMI >30 (%)	686 (39.75)	836 (47.39)	<0.0001
Waist circumference (cm), mean (SD)	93.17 (15.31)	95.62 (16.40)	<0.0001
Glomerular filtration rate (mL/min/1.73m^2), mean (SD)	94.55 (19.85)	98.51 (22.19)	<0.0001
Comorbidities (%)			
Hyperlipidemia[2]	411 (23.81)	447 (25.51)	0.24
Hypertension[3]	600 (34.76)	709 (40.08)	0.001
Diabetes mellitus[4]	211 (12.22)	268 (15.25)	0.01
Metabolic syndrome[5]	310 (17.96)	378 (21.33)	0.01
Alcohol use (g/day), median (IQR)	5.2 (15.2)	0 (13.6)	<0.0001
Smoking (%)	248 (14.37)	341 (19.84)	<0.0001
Physical activity (exercise units/week), median (IQR)	296 (358)	258 (360)	<0.0001

[1] Responded "hard" or "very hard" in response to survey question "How hard is it to get needed health services?". [2] Total cholesterol ≥240 mg/dl and/or lipid-lowering therapy. [3] Antihypertensive medication use and/or systolic blood pressure ≥140 mmHg or diastolic blood pressure ≥90 mmHg. [4] Fasting plasma glucose ≥126 mg/dl, treatment with insulin or hypoglycemic agent, 2-h post-challenge glucose ≥200 mg/dl and/or hemoglobin A1C ≥6.5%. [5] Defined using Adult Treatment Panel III criteria; SD: Standard Deviation. P-values from Chi-squared or t-tests/Wilcoxon.
